# Clinical Phenotypic Spectrum of 4095 Individuals with Down Syndrome from Text Mining of Electronic Health Records

**DOI:** 10.3390/genes12081159

**Published:** 2021-07-28

**Authors:** James Margolin Havrilla, Mengge Zhao, Cong Liu, Chunhua Weng, Ingo Helbig, Elizabeth Bhoj, Kai Wang

**Affiliations:** 1 Center for Cellular and Molecular Therapeutics, Children’s Hospital of Philadelphia, Philadelphia, PA 19104, USA; havrillaj@chop.edu (J.M.H.); zhaom3@chop.edu (M.Z.); 2 Department of Biomedical Informatics, Columbia University Irving Medical Center, New York, NY 10032, USA; cl3720@cumc.columbia.edu (C.L.); cw2384@cumc.columbia.edu (C.W.); 3 Department of Biomedical and Health Informatics, Children’s Hospital of Philadelphia, Philadelphia, PA 19104, USA; helbigi@chop.edu; 4 Division of Neurology, Children’s Hospital of Philadelphia, Philadelphia, PA 19104, USA; 5 The Epilepsy NeuroGenetics Initiative (ENGIN), Children’s Hospital of Philadelphia, Philadelphia, PA 19104, USA; 6 Department of Neurology, Perelman School of Medicine, University of Pennsylvania, Philadelphia, PA 19104, USA; 7 Division of Human Genetics, Children’s Hospital of Philadelphia, Philadelphia, PA 19104, USA; bhoje@chop.edu; 8 Department of Pediatrics, Perelman School of Medicine, University of Pennsylvania, Philadelphia, PA 19104, USA; 9 Department of Pathology and Laboratory Medicine, Perelman School of Medicine, University of Pennsylvania, Philadelphia, PA 19104, USA

**Keywords:** Down syndrome, phenotype, electronic health records, phenotypic spectrum, longitudinal study, natural language processing, text mining, large-scale

## Abstract

Human genetic disorders, such as Down syndrome, have a wide variety of clinical phenotypic presentations, and characterizing each nuanced phenotype and subtype can be difficult. In this study, we examined the electronic health records of 4095 individuals with Down syndrome at the Children’s Hospital of Philadelphia to create a method to characterize the phenotypic spectrum digitally. We extracted Human Phenotype Ontology (HPO) terms from quality-filtered patient notes using a natural language processing (NLP) approach MetaMap. We catalogued the most common HPO terms related to Down syndrome patients and compared the terms with those from a baseline population. We characterized the top 100 HPO terms by their frequencies at different ages of clinical visits and highlighted selected terms that have time-dependent distributions. We also discovered phenotypic terms that have not been significantly associated with Down syndrome, such as “Proptosis”, “Downslanted palpebral fissures”, and “Microtia”. In summary, our study demonstrated that the clinical phenotypic spectrum of individual with Mendelian diseases can be characterized through NLP-based digital phenotyping on population-scale electronic health records (EHRs).

## 1. Introduction

Human genetic disorders can have a wide variety of clinical phenotypic presentations. Text mining from electronic health records (EHRs) provides a potential avenue to systematically characterize the phenotypic spectrum of genetic disorders, when EHRs can be obtained for a large set of individuals affected with a specific disease. Down syndrome (DS) is studied here as a case study in stratifying patients affected with the same disease into phenotypic subtypes, because it is caused by an easily detectable and clearly defined genotype, yet it has highly heterogeneous clinical presentations. Down syndrome is defined by partial or complete trisomy; 21.95% of cases are represented by free trisomy 21 [1], predominantly due to a maternal meiotic nondisjunction [2]. Roughly, the other 5% of cases are due to a Robertsonian translocation and, more rarely, rea(21q21q) isochromosomes in chromosome 21 [3,4]. Lastly, only about 1% of all cases are mosaic DS [5].

While it is well-defined in genetic scope, at the same time, DS is a highly heterogeneous disease, with a wide range of phenotypic variation among affected individuals [6]. DS is the most common chromosomal disorder [7], and as prime examples of phenotypic subtypes that act as positive controls, approximately half of the patients with DS also present with congenital heart disease (CHD) [8,9,10], half of DS patients possess vision problems, and three quarters possess some form of hearing loss [10]. Hypotonia [11], apnea [12], and global developmental delay [13] are also common terms that describe many DS patients but are unlikely to describe the average healthy individual [14,15]. DS is, thus, a propitious exemplar for creating an informatics method for discovering the paramount phenotypic characteristics and comorbidities of diseases and syndromes.

Defining the spectrum of phenotypic features and characteristics in DS provides an exemplary archetype for performing similar predictions and classifications in other, well-defined phenotypes, particularly in Mendelian diseases. In order to define this spectrum, we employ the Human Phenotype Ontology (HPO) [16]. HPO was conceived as an attempt to capture discrete symptoms and phenotypic features using a hierarchical structure of phenotypic terms. This enables an approach to phenotyping where computationally derived phenotypic profiles of human diseases allow terms to be linked to similar terms in the hierarchy and disease ontologies [17,18,19], and has already been implemented as the standard for representing phenotype data by several major databases [20,21,22,23]. Natural language processing (NLP) tools can be used to extract these terms swiftly, reliably, and accurately from clinical free text and patient notes [24,25]. Digital phenotyping using this HPO-derived phenotype data has already been used to discover several causal and candidate genes for disease [18,24,26,27,28,29,30].

We describe a heretofore-unseen large-scale phenotypic dataset of 4095 individuals with DS and 7845 non-DS “baseline” subjects with various phenotypes. We utilize HPO terms extracted from DS patient notes on a patient-by-patient basis, extracting the most meaningful terms that describe DS. We further describe the age-based distribution of terms for DS individuals. We present our work in the hope of extrapolating this method to other genetic diseases.

## 2. Materials and Methods

### 2.1. Obtaining and Filtering the Clinical Notes

This study was approved by the Institutional Review Board at The Children’s Hospital of Philadelphia (CHOP) (IRB 18-015712). Only summary statistics are computed from the ensemble of clinical notes, and no identifiable information is used in this study to support its conclusions. To retrieve clinical notes on Down syndrome (DS) from the patients of CHOP, we queried patients from the Epic Clarity database maintained internally and updated daily at CHOP (with patient notes downloaded on Apr 26, 2021, and demographic data downloaded July 23, 2021) for ICD-10 code Q90* (Q90.0, Q90.1, Q90.2, Q90.9) and ICD-9 code 758.0, filtering out similar yet distinct patients with 22q.12 deletion syndrome (i.e., DiGeorge syndrome, velo-cardio-facial syndrome) possessing ICD-10 codes Q93.81 and D82.1, and ICD-9 codes 279.11 and 758.32. Many patients have incomplete notes that have less than 3000 characters or notes lacking any discernible formatting such as a medical record number (MRN), which we found to be a clear demonstration of a properly formatted note. These low-quality notes were filtered out, retaining notes determined to be of higher quality after thorough manual examination of thousands of notes. Our method for deriving the baseline population samples to simulate typical HPO term noise is described in the Supplemental file.

### 2.2. Extracting HPO Terms and Counting Them

Deriving HPO terms from notes was performed in the same way for all individuals. First, we utilized the NLP tool MetaMap (version 2020 using 2020AA UMLS USAbase) [31] to extract Unified Medical Language System (UMLS) [32] terms from the notes. We chose MetaMap as we used it in our previous work [24], and the alternative, MedLEE [33], was not freely available. After extracting the UMLS terms, we used the UMLS Metathesaurus file MRCONSO.RRF to translate UMLS CUIs with HPO equivalents to HPO IDs using the fields SAB and SDUI to match only HPO terms.

For each note, all instances of a term were counted, including duplicates to calculate the true term frequency. For document frequency, the number of documents where a term existed was counted. Inverse document frequency was just the number of documents in the whole corpus divided by this count. Lastly, for patient frequency, which is what was used for most of the results, we counted the number of patients with at least one copy of a term once per patient, to be more representative of the phenotypic spectrum of the patient population.

### 2.3. Propagation of Terms and Term Filters

As in Ganesan et al. [34], for every HPO term, we propagate all overarching parent terms by traversing down the HPO tree to its root. Therefore, if we have the term “Zonular cataract” (HP:0010920), we also add its parent, “Cataract” (HP:0000518), then its parent, “Abnormality of the lens” (HP:0000517), etc., all the way down the tree until stopping at our root term, “Phenotypic abnormality” (HP:0000118), which itself is not included because it is a redundant term that adds no information.

All HPO terms in individuals with Down syndrome are filtered out if they coincide with HPO terms possessing >5% patient frequency in our baseline cohort, for all figures.

### 2.4. Generation of Term Age Plots

We also examined the top 100 HPO terms by odds ratio and raw patient frequency and used the latter to create a top 100 term age distribution plot. The number of patient visits, or notes, containing an HPO term at all ages was used to generate the heatmap of the age-distribution plots for the top 100 terms and other select terms’ figures. All age plots use 3-month bins to obtain a snapshot of the data distribution, again as conducted by Ganesan et al. [34].

## 3. Results

### 3.1. Summarizing the Down Syndrome Data Set and Extraction of Terms

Our cohort at CHOP contains 4095 individuals with the ICD codes for Down syndrome after filtering for possible misdiagnoses. Initially, there were 784,695 notes with at least one HPO term in them, but many were false positive terms or derived from low-quality notes. After filtering by note length and format quality, we pared our set down to 3553 patients with 87,276 notes (Table 1). Our baseline cohort of 7845 patients consisted of 19,494 unfiltered notes, mainly meant to represent the usual noisy HPO terms that often appear in patient notes. Unsurprisingly, there are more clinical notes for each individual affected with DS, in comparison to the baseline cohort.

We collected demographic data (Table 2) on ethnicity and sex as well. As a result of the ICD-9/ICD-10 coded age at CHOP being used to determine diagnosis, it is unclear what the first diagnosis age was, and much of the date data were coded erroneously and have thus been omitted. The majority of patients were designated as White (65.6%), but the DS cohort had some diversity in Black (15.6%), Hispanic (4.2%), and Asian (3.2%) patients. We also had a nearly 50-50 split for Male and Female patients.

After filtering out 22q deletion patients and using MetaMap to extract UMLS terms and converting them to HPO terms, retaining duplicate terms to understand HPO term frequency accurately, we calculated the term frequency, document frequency, inverse document frequency, and patient frequency of each term (Supplementary Table S1).

### 3.2. The Phenotypic Spectrum of Down Syndrome

**Figure 1:**
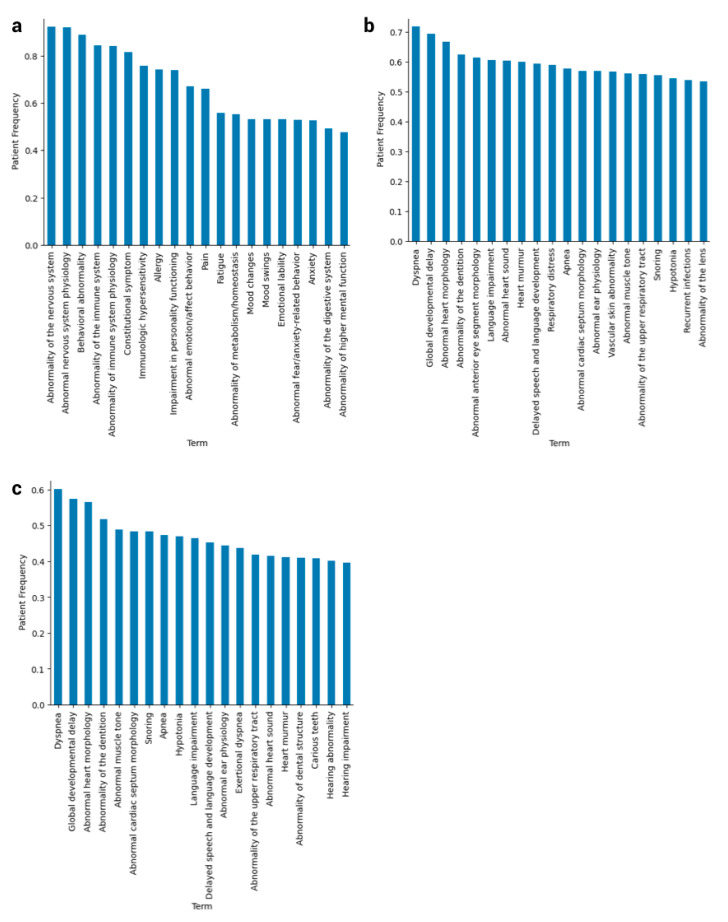
Phenotypic spectrum of HPO terms for Down syndrome and baseline patient cohorts. (**a**) These are the top 20 HPO terms from baseline patients ranked by patient frequency, or the proportion of the cohort possessing at least one instance of the term in its notes. (**b**) The top 20 HPO terms in DS patients ranked by patient frequency, after propagation up to “Phenotypic abnormality” and before filtering on note length and quality. (**c**) The top 20 HPO terms in DS patients ranked by patient frequency, after both propagation and filtering.

After testing term frequency, document frequency, and TF-IDF (term frequency × inverse document frequency) and finding that the raw spectra of terms made little sense, we sought to utilize patient frequency—the proportion of any cohort, DS or baseline, possessing at least one instance of an HPO term in any note—to glean the true spectrum of HPO terms that characterize DS patients (Figure 1). There are several notes that are copy-pasted email chains that still pass the filters, but by utilizing patient frequency instead of TF-IDF, we account for this.

We noticed that, before filtering, our HPO terms recapitulate known comorbidities for DS, such as congenital heart defects, ear and eye problems, respiratory issues, and sleep disorders at 50% patient frequency or higher (Figure 1b,c). One of the benefits of applying the filters, despite losing some of the more prominent DS terms from the top 20 at their appropriate percentages such as “Abnormality anterior eye segment morphology” (Figure 1b,c), is that more specific, rarer traits surfaced that are more characteristic of subtypes of Down syndrome.

### 3.3. Implications of New Terms Related to Down Syndrome

We found less common phenotypic features of Down syndrome, such as “Proptosis” (OR = 9.62; *p*-value = 1.90 × 10^–6^), a trait that could present in a form of leukemia associated with DS [35]; “Downslanted palpebral fissures” (OR = 10.51; *p*-value = 3.48 × 10^–6^), a term usually associated with other syndromes, as upslanted fissures are more characteristic of DS [36]; and examples such as “Hypoplasia of penis” (OR = 9.54; *p*-value = 2.39 × 10^–6^) and “Microtia” (OR = 12.78; *p*-value = 7.11 × 10^–6^), which we could not find in the DS-related literature. We acknowledge that that OR measures should only be used as a rough reference, given the highly unbalanced number of notes per individual between the DS case cohort and the baseline cohort (See Supplementary file).

We have 4748 HPO terms that were used to describe Down syndrome patients sorted by patient frequency (Supplementary Table S2). We have also extracted 1157 HPO terms compared between DS cases and our baseline cohort that we use to represent mock controls that are sorted by odds based on document frequency and only have *p*-values that below the Bonferroni 5% FDR adjusted *p*-value cutoff (Supplementary Figure S1, Supplementary Table S3). These files contain many more such instances of Down syndrome terms that are unique to this study, while, at the same time, replicate previous results, containing mostly heart terms at our very highest OR and *p*-values such as “Holosystolic murmur” (OR = 256.60; *p*-value = 0.04), “Left-to-right shunt” (OR = 5.94 × 10^–6^; *p*-value = 8.45 × 10^–6^), and Myeloproliferative disorder (OR = 159.20; *p*-value = 4.17 × 10^–6^).

### 3.4. Longitudinal Distribution of HPO Terms in Down Syndrome

**Figure 2:**
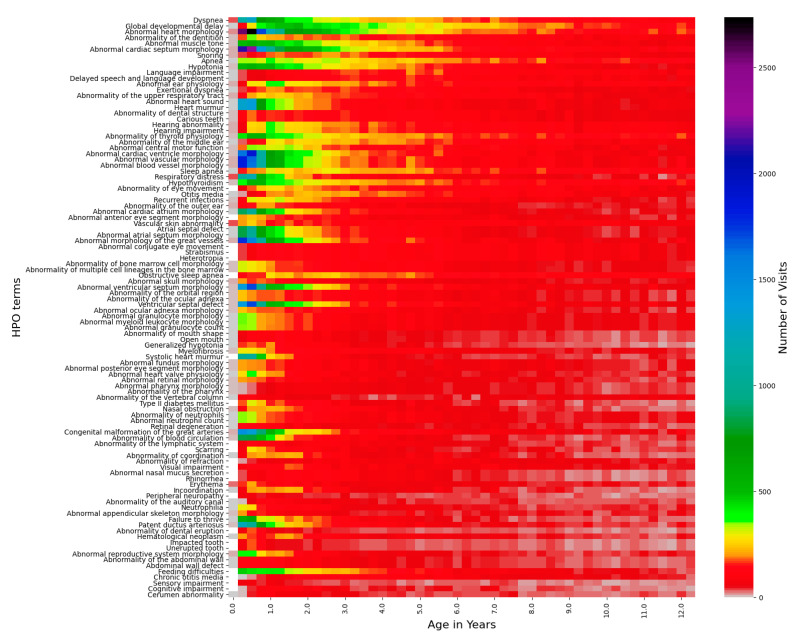
Top 100 HPO terms age distributions for the Down syndrome cohort. The number of visits with a certain age within a 3-month bin is represented by the color bar on the right, the top 100 HPO terms are listed on the left-side *y*-axis, and the age in years from 0–12.5 is listed on the *x*-axis.

**Figure 3:**
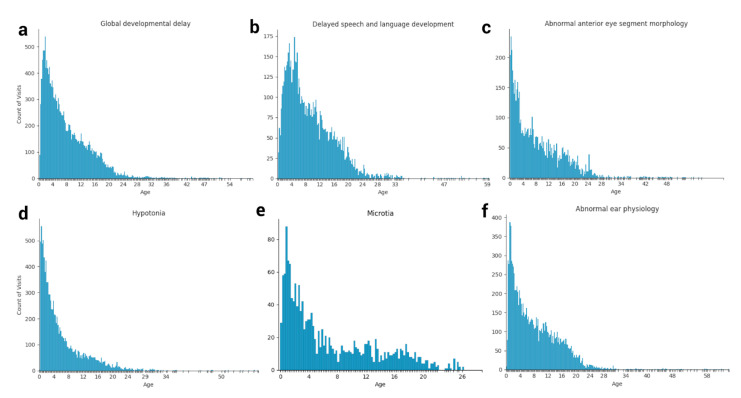
Snapshots of select HPO term age distributions for the Down syndrome cohort. The occurrence of various HPO terms at each patient visit reflects the longitudinal distribution (age in years) of each feature in Down syndrome, including (**a**) Global developmental delay, (**b**) Delayed speech and language development, (**c**) Abnormal anterior eye segment morphology, (**d**) Hypotonia, (**e**) Microtia, and (**f**) Abnormal ear physiology.

The longitudinal distribution of HPO terms in DS patients provides a snapshot of the typical diagnostic ages for terms such as “Delayed speech and language development”, which cannot be diagnosed until the average child can speak at around 2–4 years of age [37] (Figure 2 and Figure 3, Supplementary Figure S2). Other terms, such as “Abnormal heart morphology” or its child terms are diagnosed extremely early on in the patients’ lives, typically in the first 3–12 months. Lastly, terms such as hypothyroidism or sleep apnea are diagnosed steadily throughout childhood with no particular favoritism given to a specific age of diagnosis. Ear abnormalities take some time to diagnose, usually a year or so, but more often than not, eye problems and hypotonia are diagnosed right away (Figure 3).

## 4. Discussion

With this study, which is currently among the largest phenotypic studies of Down syndrome to date, we have created a basic framework for filtering raw clinical text, extracting accurate ontological terms from those notes, and analyzing the phenotypic spectrum of a relatively common genetic syndrome. We have stratified HPO terms by patient frequency, and a document frequency-based odds ratio, and used the top 100 terms to understand the time-based distribution of HPO term data by patient visits, as well as the specific minimal age at which certain terms can be used to describe a patient. We also discovered some unique terms that have not been previously associated with Down syndrome and may be specific to this CHOP dataset.

However, this study has several limitations that need to be addressed. First, we need to obtain more information on the quality of the notes. When obtaining notes for a disorder such as Down syndrome, patients come into the hospital for all manner of visits, such as urology, cardiology, oncology, rheumatology, etc. Each department has a vastly different note format, varying significantly in quality, style, and length, both within and between departments. Our best attempt at filtering was a simple one meant to deal with the general lack of understanding about the various types of notes. Collaboration with physicians who have intimate knowledge of the clinical data specific to their department or cohort will allow us to obtain the most high-quality notes and create clusters of subgroups for the relevant phenotype. Due to the low signal-to-noise ratio that still exists largely due to this factor, we could not create useful clusters of patient subgroups.

Second, the methodology for obtaining our baseline patients could be further improved. We randomly grabbed patients with different reasons for visits to obtain a good distribution of phenotype, but as a result we had a nearly 5 to 1 ratio of DS notes to baseline notes for almost double the amount of baseline patients. This is quite unbalanced. It may be better to use a different, larger cohort as a true control background such as general patients coming for allergies and/or diseases that are extremely common and do not affect the health of the patient very much. Nevertheless, such a control set is still unlikely to address the issue that generally disease-free individuals may not visit many specialty departments or clinical geneticists or have as many detailed phenotypic descriptions as Down syndrome.

Our NLP pipeline is also generally simple. We only use MetaMap and, while we tested other tools and found it to be the best in terms of performance, we could create our own ensemble method, as a combination of MetaMap’s results and several other tools, for averaging the extracted terms. There are several instances where MetaMap annotated UMLS terms that were not in the notes at all, which were then translated into HPO terms. These became largely background noise but could be affecting the signal-to-noise ratio of the phenotypic information data on DS.

In the future, we would like to improve upon this pipeline and use a secondary validation site to validate these results and see if we can simultaneously increase the signal-to-noise ratio. We will also seek collaboration with other physicians and genetic counselors at CHOP to assist us in interpreting the notes of this and future phenotypes to ensure that we can further raise the quality and create a reproducible and fast pipeline for analyzing the phenotypic data of common diseases. After creating this pipeline, we would like to further subtype and group patients in order to better predict comorbidities and future diseases or phenotypes that may befall a patient, to ensure that preventative medicine can be used to combat them.

## 5. Conclusions

Our analysis profiled the spectrum of phenotypic features in patients with DS in the form of the HPO standardized terminology. We demonstrated that an NLP-based approach for extracting HPO terms has value for characterizing DS as a disease with subtypes and provided a quantitative measure of the HPO terms to facilitate future construction of phenotype-based patient sub-classification models, which will allow for clinical decision support and learning health systems. We hope that other researchers can utilize our methodology to characterize other common diseases with phenotypic subtypes and understand the time-based distribution of the phenotypic feature data.

## Figures and Tables

**Table 1:** Population numbers for Down syndrome and baseline patient cohorts. **#** This details the number of patients, their notes, and notes after filtering out short and low-quality notes.

Dataset	# Patients	# Notes (with ≥1 HPO Terms)
DS cases	4095	784,695
DS (filtered)	3553	87,276
Baseline	7845	19,494

**Table 2:** Demographics for Down syndrome patient cohort. Gender and ethnicity values are Epic-coded. Ethnicity values are not mutually exclusive, and each row combined does not add up to the total number of patients.

Category		
Sex	Male	2145
	Female	1950
Ethnicity	White	2685
	Asian	130
	American Indian	9
	Native Hawaiian/Pacific Islander	15
	Black	635
	Hispanic	170
	Other	684
	Unknown	56

## Data Availability

The code can be found at https://github.com/WGLab/Down-Syndrome-Analysis. The individual level data cannot be made available to protect patient privacy. However, all deidentified summary level data are available in the supplement.

## Supplementary Materials

The following are available online at https://www.mdpi.com/article/10.3390/genes12081159/s1. Supplementary file: Methods and Results. Supplementary Table S1: Describes the contingency table used for the Fisher’s exact test and odds ratio calculations. Supplementary Table S2: Contains all 4748 HPO terms from the filtered DS patient notes sorted by patient frequency, with term, document, patient frequency data, and TF-IDF. HPO Terms sorted by patient frequency. TC is term count, DC is document count, PC is patient count, IDF is inverse document frequency, TF-IDF is term frequency times inverse document frequency, and PF is patient frequency, by which the table is sorted. Supplementary Table S3: Contains 1157 HPO terms from DS and mock controls combined sorted by odds ratio, calculated using document frequency. Contains OR and *p*-values for HPO terms filtered on Bonferroni corrected FDR cutoff of 5%. Supplementary Figure S1: The top 20 HPO terms, ranked by odds ratio of patient occurrence between DS cases and mock controls, an expansion of Figure 1. Supplementary Figure S2: An expanded version of Figure 2 for all age values.
