# Supplementary material for: Clinical Phenotypic Spectrum of 4095 Individuals with Down Syndrome from Text Mining of Electronic Health Records
Source: Genes (Basel). 2021 Jul 28;12(8):1159. doi: 10.3390/genes12081159 (PMC8393657; doi:10.3390/genes12081159)
Supplement: Supplementary file 1 [file genes-12-01159-s001.zip › supplementary file & table S1.pdf]

Methods and Results

1. Obtaining and filtering the patient notes for mock controls

The 7,845 individual baseline cohort, meant to represent noise in patient data, is also our mock "control" cohort. Their 19,494 unfiltered notes came from various phenotypes and consisted of patients without the ICD codes for DS, and without circulatory disorders, as indicated by the lack of ICD-10 codes beginning with "I" or an ICD-9 code in the range between 390 and 459. A small proportion of control patients did have cardiovascular HPO terms despite this filter, and the majority of controls have no mental and behavioral disorders, or ICD codes beginning with an "F". In genetic studies, *de novo* mutations in unaffected siblings of autism probands have been used to simulate benign variants [1]. Similarly, we chose random patients with some psychiatric notes but no psychiatric disorders to simulate healthy patient notes, to obtain a randomly distributed assortment of term data. Since individuals affected with Down syndrome are more likely to visit many more speciality departments (cardiology, neurology, etc) than the baseline cohort, we found that the average number of notes in the baseline cohort is much lower than in the Down syndrome cohort, which is a factor that should be considered when interpreting the results.

2. Comparing mock controls to cases

We compared and contrasted the phenotypic spectrum of control HPO terms from DS case terms and ranked the terms by odds ratio as compared to mock controls.

3. Odds ratio comparison of terms

A comparison of case and control HPO terms significantly enriched in DS patients by Fisher's exact test (ORs discussed in the Results are all Bonferroni-corrected significant) and a simple odds ratio calculation was performed as described in the table and formulae 1 and 2 below. To calculate the odds ratios we used document frequency instead of patient frequency, to compensate for the data imbalance in notes between DS cases and controls (a ~4.5 to 1 note imbalance after filtering). Odds ratios calculated are thus high both because they are based on patient count, and because terms >5% in patient frequency in our mock "controls" are filtered from DS results.

The Bonferroni correction divides our p-value cutoff of $\alpha=0.05$ by the number of tests, in this case, one test per HPO term evaluated (5,282) for an odds ratio between case and controls (Results in **Supplementary Table 3**).

	DS	Control
**Patients with Term**	Dw	Hw
**Patients without Term**	Do	Ho

Supplementary Table S1. Explanation of odds ratio parameters for phenotypic term enrichment. This was used to calculate terms enriched in a specific cluster over other clusters as well as in cases over controls.

The formula for calculating the p-value [2] is:

$$p = \frac{(D_w+H_w)!(D_o+H_o)!(D_w+D_o)!(H_w+H_o)!}{D_w!H_w!D_o!H_o!n!} \quad (1)$$

and the formula for the odds ratio is:

$$OR = \frac{D_w/H_w}{D_o/H_o}. \quad (2)$$

Top 20 ranked terms by odds ratio

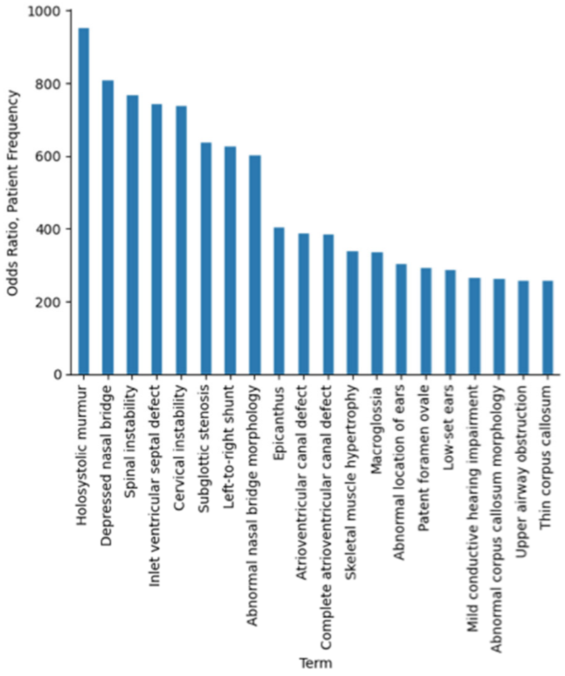

Supplementary Figure S1. The top 20 HPO terms, ranked by odds ratio of patient occurrence between DS cases and mock controls.

Longitudinal study of HPO terms for DS patients with no age cutoff

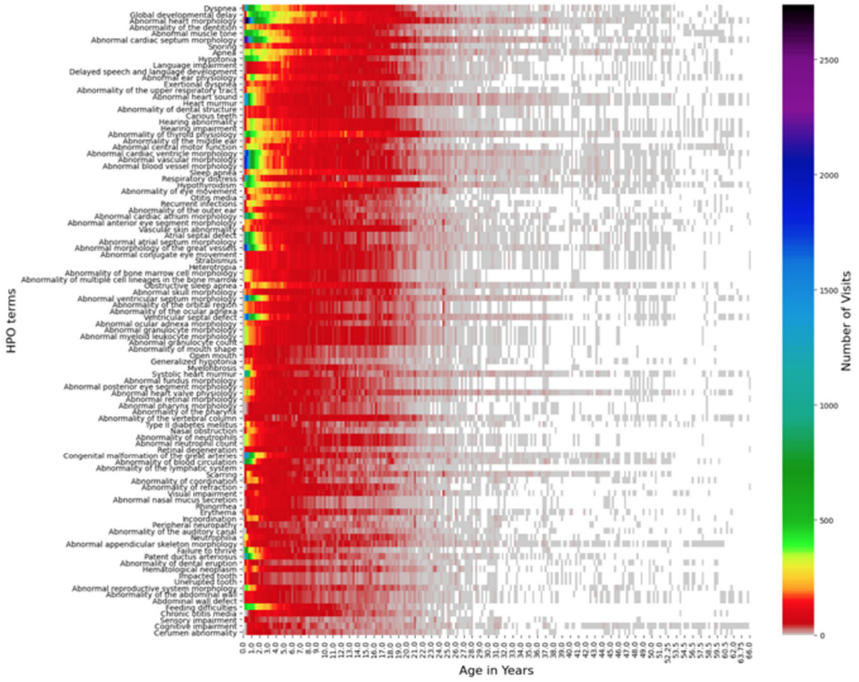

Supplementary Figure S2. The top 100 HPO terms, ranked by patient frequency for DS cases without an age cutoff.

References

1. Havrilla, J.M.; Pedersen, B.S.; Layer, R.M.; Quinlan, A.R. A Map of Constrained Coding Regions in the Human Genome. *Nat. Genet.* **2019**, *51*, 88–95.
2. Jafari, M.; Ansari-Pour, N. Why, When and How to Adjust Your P Values? *Cell J.* **2019**, *20*, 604–607.
