# Supplementary figures and images for: Clinical Phenotypic Spectrum of 4095 Individuals with Down Syndrome from Text Mining of Electronic Health Records
Source: Genes (Basel). 2021 Jul 28;12(8):1159. doi: 10.3390/genes12081159 (PMC8393657; doi:10.3390/genes12081159)

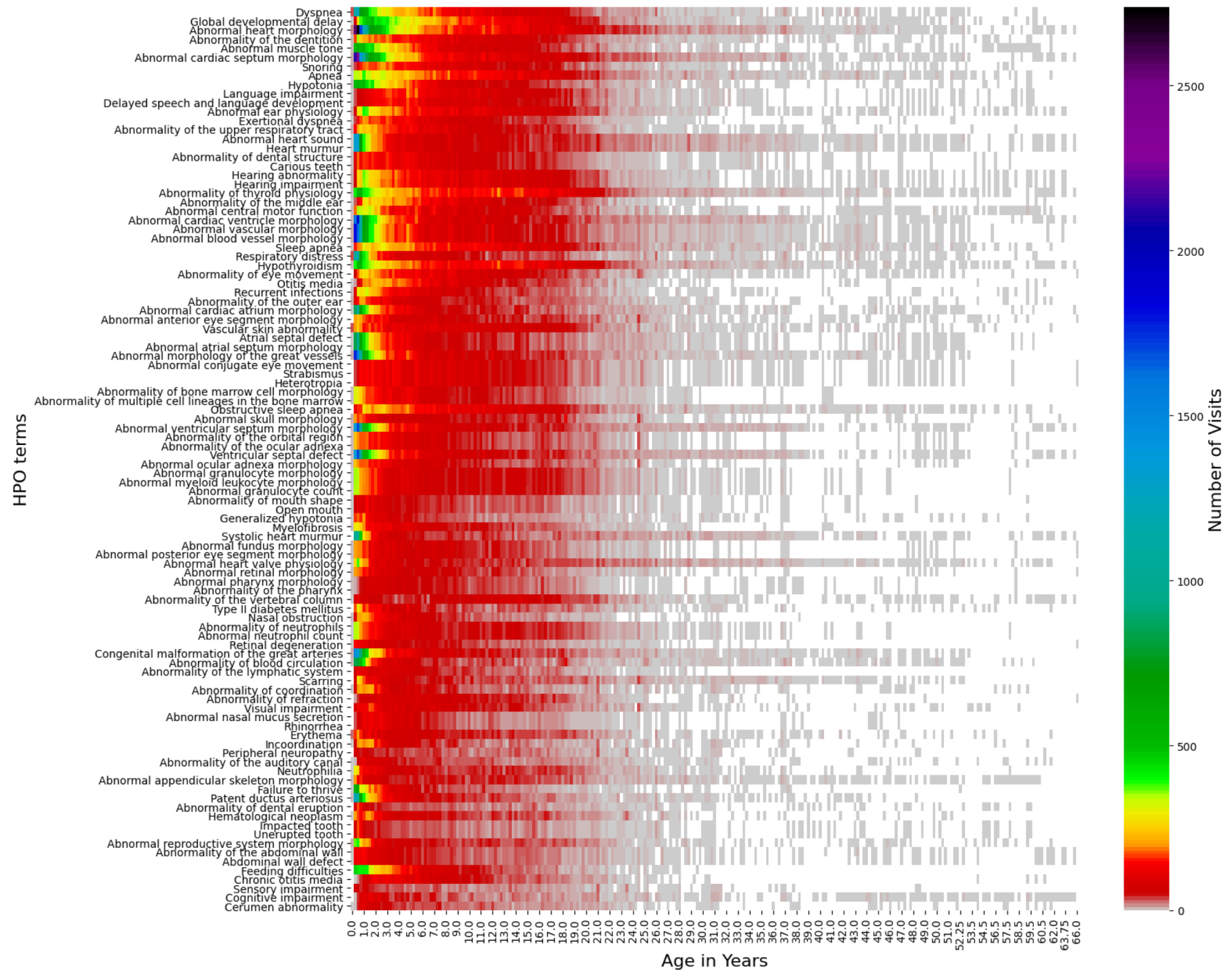

Supplement: Supplementary file 1 [file genes-12-01159-s001.zip › Figure S.pdf]

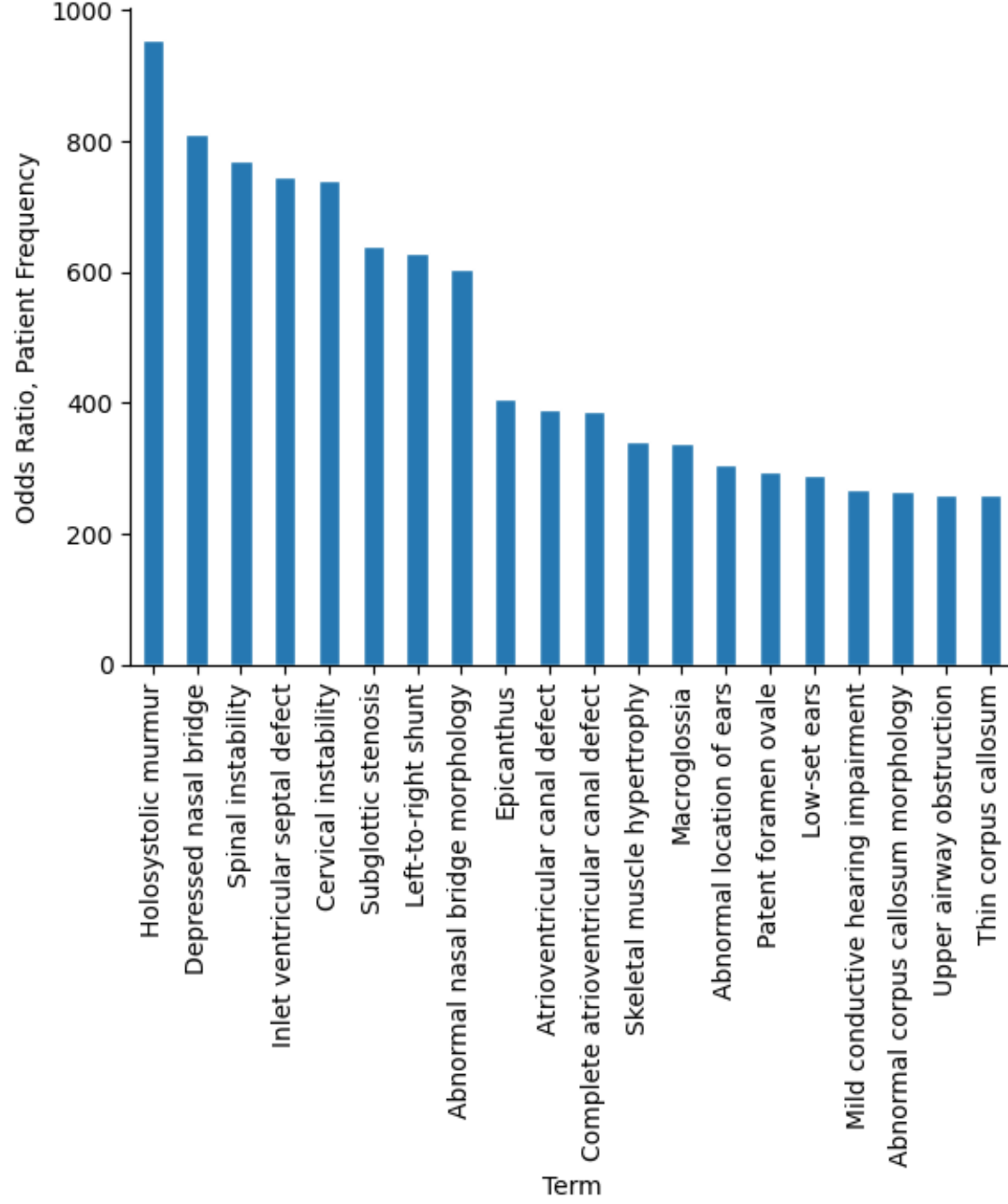

Supplement: Supplementary file 1 [file genes-12-01159-s001.zip › Figure S1.pdf]
